# Variation in the Branches of Arch of Aorta in Patients Undergoing Contrast Enhanced Multidetector Computed Tomography in a Tertiary Center: A Descriptive Cross-sectional Study

**DOI:** 10.31729/jnma.8605

**Published:** 2024-06-30

**Authors:** Prakash Kayastha, Sharma Paudel, Nikesh Bista, Binaya Adhikari, Shashi Shekhar Singh, Shailendra Katwal

**Affiliations:** 1 Department of Radiology and Imaging, Tribhuvan University Teaching Hospital, Maharajgunj, Kathmandu, Nepal; 2 Department of Radiology and Imaging, Damak Hospital, Jhapa, Nepal; 3 Department of Radiology and Imaging, National Trauma Center, Mahaboudha, Kathmandu, Nepal

**Keywords:** *arch of aorta*, *cardiovascular*, *ethnic groups*, *MDCT*, *surgical procedure*

## Abstract

**Introduction:**

Variations in the branching pattern of the Arch of Aorta (AoA) are common in patients undergoing contrast-enhanced Multidetector Computed Tomography, the identification of which is crucial in managing patients undergoing cardiovascular/neck surgeries and interventions.

**Methods:**

This descriptive cross-sectional study involved 513 patients who were sent to the Department of Radiology for evaluation of various pathologies of chest and neck between August 2018 and July 2019. After approval from the Institutional Review Committee {Reference No: 11(6-11) E2/075/076}, contrast-enhanced computed tomography images were evaluated with variations in branches of the left-sided arch of the aorta and symptoms associated.

**Results:**

Variations in branches of the arch of aorta were seen in 69 (13.45%; 95% CI: 10.60%-16.71%) of cases, left common carotid artery and brachiocephalic trunk having common origin or common trunk was 51 (9.94%). The mean age was 52.4±20 years (Range 3 months to 92 years) with male to female ratio of 1.3:1.

**Conclusions:**

Contrast-enhanced computed tomography is the modality of choice for the detection of the variations in branches of AoA, recognition of which is crucial in vascular intervention and surgical procedures to reduce the postoperative morbidity and mortality of the patients.

## INTRODUCTION

The wide spectrum of variation of great arteries at the thorax is well recognized.^1-4^ Variations in the branching of the Arch of Aorta (AoA) typically manifest asymptomatically and are often incidentally detected during radiological evaluations for unrelated reasons.^5^ The shared point of origin of brachiocephalic trunk (BCT) and left common carotid artery (LCCA) may correlate with congenital cardiac anomalies and coronary artery irregularities.^6-7^

The atypical emergence of the left vertebral artery (LVA) directly from the AoA heightens the risk during head and neck surgical interventions.^8-10^ Computed Tomography (CT) adeptly identifies congenital anomalies of the aortic arch and major vessels, crucial for informed surgical decisions in the neck and thoracic regions.^1^ Notably, only two cadaveric studies in Nepal have explored variations in the aortic arch's branching pattern.^11^

Our study aimed to delineate such variations in our population using contrast-enhanced Computed Tomography (CECT), elucidating their prevalence and patterns for clinical guidance.

## METHODS

A prospective study was conducted involving patients referred to the Department of Radiology and Imaging at Tribhuvan University Teaching Hospital (TUTH) by clinicians from TUTH as well as other hospitals and clinics. These referrals were made for the purpose of contrast enhanced CT (CECT) scans of the chest and neck, which also encompassed imaging of the aortic arch, pulmonary arteries, and carotid arteries. The study spanned from August 2018 to September 2019. Scans were conducted utilizing the Siemens Somatom Definition AS+ 128-slice multidetector computed tomography (MDCT) scanner available in the Department of Radiology and Imaging. Ethical clearance was secured from the institutional review board of the Institute of Medicine {Reference No: 11(6-11) E2/075/076} and informed consent was obtained from all participating subjects.

Sample size was calculated based on Cochrane formula n = Z^2^pq/e^2^, at 95% level of significance and allowable error (e) at 5%.

The tabulated value of Z at 95% level of significance is 1.96, Z^2^ = (1.96)^2^ = 3.84; prevalence(p) = 20%.^12^, q = 100-p = 20; e^2^ = 25. The calculated minimum sample size was 246, however, a total 513 subjects were included in the study. Participants were enrolled using a convenience sampling technique where consecutive samples were taken.

The study included patients who underwent CECT scans of the chest and neck at TUTH within a one-year timeframe for various medical reasons. Patients with vasculitis affecting large vessels, chest and neck pathologies grossly distorting vascular anatomy of branches of the aortic arch and those not giving consent were excluded from the study.

Aortic arch branching pattern were divided into five different types as in a study of Karacan A. et al.^5^ In type I pattern Brachiocephalic trunk (BCT) emerged as the first branch, followed by the left common carotid artery (LCCA) as the second branch, and the left subclavian artery (LSCA) as the third branch (from right to left). Concurrently, the right common carotid artery (RCCA) and the right subclavian artery (RSCA) were observed as branches originating from the BCT, while the right vertebral artery (RVA) and the left vertebral artery (LVA) arose from the RSCA and LSCA, respectively. In type II branching pattern, RCCA and BCT either had a common trunk arising from AoA or had a single origin in AoA. In type III pattern, the LVA was arising directly from AoA between LCCA and LSCA as the third branch of AoA (from right to left). In type IV pattern, RSCA arised as the last branch of AoA (from right to left) after LSCA instead of its origin from BCT, as found in type I. Type V pattern was described where type IV pattern was seen in combination with truncus bicaroticus, i.e., RCCA and LCCA had a common trunk.

Type I branching pattern was considered as the normal configuration. All other branching patterns (type II to V) were regarded as anatomical variants.

The CECT scans were meticulously assessed utilizing multi-planar reconstruction images alongside threedimensional reformatted images employing maximum intensity projections and volume rendering techniques. Any deviations observed in the arterial structure as well as any vascular pathologies visualized in the images were diligently documented and analyzed. The collected data analysed through statistical analysis using the Statistical Package for Social Sciences (SPSS).

## RESULTS

The mean age of the sampled population was 52.4±20 years (3 months to 92 years). The age group of 61 to 80 acounted for 199 cases (38.79%), (Table 1).

**Table 1:** Age distribution of the patients (n = 513).

Variables	n (%)
Age in years	
0-20	44 (8.57)
21-40	108 (21.05)
41-60	144 (28.07)
61-80	199 (38.79)
>80	18 (3.50)

The variations in branches of arch of aorta were seen in 69(13.45%; 95% CI: 10.60%-16.71% ) of cases, left common carotid artery and brachiocephalic trunk having common origin or common trunk was 51 (9.94%), (Table 2).

**Table 2:** Various aortic arch branching patterns (n = 513).

Type of Branching Pattern	n (%)
Type I	444 (86.55)
Type II	51 (9.94)
Type III	14 (2.73)
Type IV	3 (0.59)
Type V	1 (0.19)

Except for one case of dysphagia lusoria present in type IV variant, there was no significant symptom associated with any other variant of branches of AoA in our study.

## DISCUSSION

Variations in the branching patterns of the aortic arch (AoA) are quite prevalent. Recognition of such variations holds significance for intervention radiologists, cardiothoracic and neck surgeons, particularly in devising surgical strategies for patients afflicted with various pathologies affecting the chest and neck. While Digital Subtraction Angiography (DSA) stands as the gold standard for assessing patients with vascular pathologies and for delineating the branching patterns and trajectories of arteries, contrast-enhanced Multidetector Computed Tomography (MDCT) is frequently employed due to its lesser invasiveness and its capacity to furnish a three-dimensional representation of the vasculature, along with the precise delineation of the relationship between vessels and pathologies.

In our examination of contrast-enhanced MDCT images encompassing 513 cases, 13.45% exhibited variations in the branching pattern of the AoA, a figure marginally lower than the findings of a cadaveric study conducted by C. Bhattarai et al., comprising 85 cases from the Nepalese populace, where 20% of cases displayed such variations.^11^ Nonetheless, a computed tomography (CT) examination-based investigation by Muller et al., involving 2033 cases from the German population, revealed variant anatomy in 13.3% of cases, aligning closely with our findings.^13^ Popieluszko et al., through a systematic review and meta-analysis encompassing 51 cadaveric and imaging-based studies totaling 23,882 cases, determined a prevalence of variant anatomy at 19.1% across all studies and 13.1% within the Asian population subset.^12^ Remarkably, this prevalence mirrors our own finding of 13.45% variant anatomy.

The most frequently encountered variant in our study was the left common carotid artery (LCCA) and brachiocephalic trunk (BCT) originating from a common point or trunk, denoted as the type II branching pattern in our investigation, observed in 9.94% of cases. This figure slightly deviates from the 12.9% prevalence identified in the cadaveric study conducted on the Nepalese population by C. Bhattarai et al.^11^ Our observation of this branching pattern exceeds that of Muller et al., where it was noted in 8% of cases, and surpasses the prevalence reported by Popieluszko et al. for the Asian population, which stood at 7.4%.^12,13^

The next variant found in our study was LVA arising directly from AoA between LCCA and LSCA, described in our study as type III branching pattern, which was seen in 2.73 % of cases. This finding was lower than studies done by C Bhattarai et al (7%), Muller et al (4.2%) and Asian subgroups of metaanalysis by Popieluszko et al (3.5%).^11-13^

Another variant found in our study was aberrant RSCA where RSCA arises as the last branch of AoA after LSCA and traverses behind the trachea and esophagus towards the right arm. This branching pattern was referred to as Type IV pattern in our study and was found in 0.59% of cases, which was higher than study by C Bhattarai et al (0%) and Asian subgroup of metaanalysis by Popieluszko et al (0.5%), and similar to study by Muller et al (1%).^11-13^ Inability to detect this branching pattern in study by C Bhattarai et al could be due to less number of cases (85 compared to 513 in our study) and relatively lower prevalence of this branching pattern.

Our study also found 1 case (0.19%) of a variant in which aberrant RSCA was seen in combination with common origin of RCCA and LCCA, referred to as Type V branching pattern in our study. This rare variant was not found by C Bhattarai et al and Muller et al in their study and was reported only in few studies in metaanalysis by Popieluszko et al.^11-13^

The other less common variations like combination of Type I and III patterns and branching pattern where RCCA and LCCA have common origin from AoA, seen in 0.4% and 0.3% cases respectively in meta-analysis by Popieluszko et al,^12^ was not found in our study.

Like other studies, all types of branching pattern in our study had no significant symptom related to variation in branching pattern of AoA, except for 1 out of 4 cases (25%) of type IV variant, where dysphagia was a major complaint. This finding of symptom associated with type IV branching pattern was, however, lower than that found in study by Donnelly et al where symptomatic cases were as high as 55.5% and included symptoms of tracheal compression as well.^14^

However, significant differences in occurrence of variant anatomy of branching pattern of AoA among ethnic groups have been reported in study by Natsis et al^3^, where variants were more common in black people.

Metaanalysis by Popieluszko et al also found out that Type II branching pattern was seen in 26.8% of the African population (compared to 9.9% in our study).^12^ In comparison to the study conducted by Pandalai et al.^15^ in the South Indian population, our study observed a slightly higher prevalence of type IV branching pattern (0.18% vs. 0.60%). Goray et al. outlined the occurrence of anomalous bilateral vertebral arteries originating from the aortic arch beyond the left subclavian artery.^16^ However, our investigation did not encounter this particular variation.

## CONCLUSIONS

Contrast-enhanced MDCT is preferred for assessing chest and neck pathology. Variation in the aortic arch's branching pattern is common among patients under evaluation for such issues and should be specifically reported to guide interventional and surgical planning.
